# Safety of tubal ligation by minilaparotomy provided by clinical officers versus assistant medical officers: study protocol for a noninferiority randomized controlled trial in Tanzanian women

**DOI:** 10.1186/s13063-017-2235-6

**Published:** 2017-10-26

**Authors:** Mark A. Barone, Zuhura Mbuguni, Japhet Ominde Achola, Carmela Cordero, Joseph Kanama, Projestine S. Muganyizi, Jamilla Mwanga, Caitlin Shannon, Leopold Tibyehabwa

**Affiliations:** 1 EngenderHealth, 440 Ninth Avenue, 12th Floor, New York, NY 10001 USA; 2 Ministry of Health, Community Development, Gender, Elderly, and Children, 6 Samora Machel Avenue, P.O. Box 9083, 11478 Dar es Salaam, Tanzania; 3 EngenderHealth Kenya, Trance Towers, 7th Floor, Tsavo Road, Off Mombasa Road, P.O. Box 57964-00200, Nairobi, Kenya; 4 RESPOND Tanzania Project, EngenderHealth Tanzania, Plot No. 254, Mwai Kibaki Road/Kiko Avenue, P.O. Box 78167, Dar es Salaam, Tanzania; 5 Association of Gynecologists and Obstetricians of Tanzania, P.O. Box 65222, Dar es Salaam, Tanzania

**Keywords:** Task shifting, Tubal ligation, Minilaparotomy, Noninferiority randomized controlled trial

## Abstract

**Background:**

Female sterilization by tubal ligation is a safe, extremely effective, and permanent way to limit childbearing. It is the most popular modern contraceptive method worldwide. The simplest way to provide tubal ligation is by a procedure called *minilaparotomy*, generally performed with the client under local anesthesia with systemic sedation and analgesia. In Tanzania, unmet need for family planning is high and has declined little in the past decade. Access to tubal ligation is limited throughout the country, in large part because of a lack of trained providers. Clinical officers (COs) are midlevel health workers who provide diagnosis, treatment, and minor surgeries. They are more prevalent than physicians in poorer and rural communities. Task shifting—the delegation of some tasks to less-specialized health workers, including task shifting of surgical procedures to midlevel cadres—has improved access to lifesaving interventions in resource-limited settings. It is a cost-effective way to address shortages of physicians, increasing access to services. The primary objective of this trial is to establish whether the safety of tubal ligation by minilaparotomy provided by COs is noninferior to the safety of tubal ligation by minilaparotomy provided by physicians (assistant medical officers [AMOs]), as measured by rates of major adverse events (AEs) during the procedure and through 42 days of follow-up.

**Methods/design:**

In this facility-based, multicenter, noninferiority randomized controlled trial, we are comparing the safety of tubal ligation by minilaparotomy performed by trained COs versus by trained AMOs. The primary outcome is safety, defined by the overall rate of major AEs occurring during the minilaparotomy procedure and through 42 days of follow-up. The trial will be conducted among 1970 women 18 years of age or older presenting for tubal ligation at 7 study sites in northern Tanzania.

**Discussion:**

If no major safety issues are identified, the data from this trial may facilitate changes in the Tanzanian government’s regulations, allowing appropriately trained COs to provide tubal ligation by minilaparotomy. Positive findings may have broader implications. Task shifting to provide long-acting contraceptives, if proven safe, may be an effective approach to increasing contraceptive access in low- and middle-income countries.

**Trial registration:**

ClinicalTrials.gov, NCT02944149. Registered on 14 October 2016.

**Electronic supplementary material:**

The online version of this article (doi:10.1186/s13063-017-2235-6) contains supplementary material, which is available to authorized users.

## Background

Female sterilization by tubal ligation is a safe, extremely effective, and permanent way to limit childbearing, and it is the single most popular modern contraceptive method worldwide [1]. The simplest way to provide tubal ligation is through a procedure called *minilaparotomy*, which is generally performed with the client under local anesthesia with systemic sedation and analgesia. According to the World Health Organization (WHO), tubal ligation by minilaparotomy is a minor surgery that can be performed in resource-limited settings on an outpatient basis with low risk of complications [2, 3]. Although data are limited and published reports are for the most part several decades old, complication rates of 0.7–3.4% following tubal ligation by minilaparotomy performed by physicians have been reported in several countries in Africa [4–9].

Among currently married Tanzanian women 15–49 years old, 25.7% do not want to have any more children, whereas 3.4% have had a tubal ligation [10]. Although modern contraceptive use has steadily increased over the last decade, from 20% in 2004–2005 to 27% in 2010 and 32% in 2015–2016, unmet need for family planning has remained steady at 22–24% since 1999 [10]. Use of tubal ligation among currently married women has more than doubled, from 1.6% in the early 1990s to 3.4% today; however, it represents only about 11% of all contraceptive use, despite the large numbers of women who desire no more children [10].

Despite a high demand for modern contraceptives, access to tubal ligation in Tanzania is limited, with 58% of hospitals and 38% of health centers offering tubal ligation services. (In some cases, only counseling is provided.) Availability is roughly evenly divided between government, private for-profit, and faith-based facilities [11]. The government of Tanzania (GOT) recognizes that most health facilities in the country are understaffed, with the greatest need in rural areas, and that a lack of trained providers significantly limits the availability of services, including tubal ligation [12]. Clinical officers (COs) are midlevel providers who diagnose and treat illnesses and perform minor surgeries. They outnumber physicians, both medical officers (MOs) and assistant medical officers (AMOs), by a 2:1 ratio, and they are more prevalent in rural communities [11].

Task shifting, described as delegation or shifting of some tasks to less-specialized health workers, is one method used to expand the health workforce [13, 14]. Task shifting of surgical procedures to midlevel cadres, including COs, has improved access to lifesaving interventions in resource-limited settings and is considered a safe and cost-effective way to address physician shortages [15]. Two recent systematic reviews of older studies, as well as two recent small-scale studies in Uganda and Malawi, suggest that task-shifting tubal ligation via minilaparotomy to nonphysician clinicians is safe, although available evidence is limited and well-designed clinical trials are needed to definitively demonstrate safety, efficacy, and acceptability of task-shifting the procedure to midlevel providers [16–19]. WHO guidelines on optimizing health workers’ roles for maternal and newborn health include COs among those considered competent to provide tubal ligation; however, the recommendation was not based on a review of evidence [20].

Task shifting of tubal ligation by minilaparotomy to COs would increase access to tubal ligation for many Tanzanian women who are most in need and is of interest to the GOT as a way to assist in meeting the needs of the growing numbers of women seeking permanent contraception. With this in mind, the primary objective of the present study is to establish whether the safety of tubal ligation by minilaparotomy, as measured by rates of major adverse events (AEs) during study participation, when provided by trained COs is noninferior to the safety of the procedure when provided by trained AMOs.

## Methods/design

**Fig. 1:**
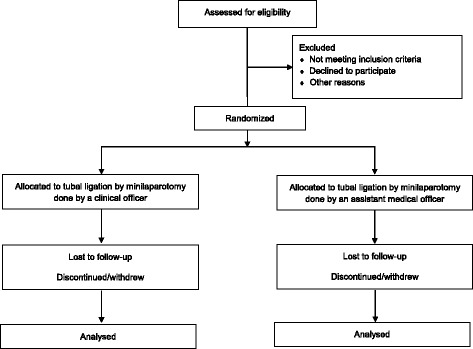
Trial profile

This study is a noninferiority randomized controlled trial (RCT) comparing the safety of tubal ligation by minilaparotomy conducted by trained COs and by trained AMOs in Tanzania. We will randomize a total of 1970 women 18 years of age and older presenting at 7 sites for tubal ligation in a 1:1 ratio to have the procedure done by a CO or an AMO. In addition to the screening and enrollment/tubal ligation visit, there will be three scheduled follow-up visits at 3, 7, and 42 days postsurgery. Figure 1 depicts the flow of study participants. We have included a Standard Protocol Items: Recommendations for Interventional Trials (SPIRIT) checklist in Additional file 1.

### Intervention

The study intervention is tubal ligation via minilaparotomy conducted by a CO in the intervention arm and by an AMO is the control arm. To ensure adequate skills to perform minilaparotomy, COs and AMOs (none of whom had prior experience performing tubal ligation by minilaparotomy) participated in competency-based training according to GOT guidelines and standards prior to the start of the study [21].

### Outcome measures

The primary objective is to establish whether the safety of tubal ligation by minilaparotomy as provided by COs is noninferior to the safety of tubal ligation by minilaparotomy as provided by AMOs. The primary study outcome is safety, defined by the overall rate of major AEs observed during the procedure and through 42 days of follow-up. We will compare the rate of major AEs observed following procedures conducted by COs with that of procedures performed by AMOs. Major AEs include the following:

1. Injuries to abdominal viscera, pelvic abscess, or severe peritonitis leading to unintended major surgery
2. Severe intra- or immediate postoperative hemorrhage requiring blood transfusion
3. Febrile morbidity (oral temperature > 38 °C on at least 2 postoperative days, excluding the first 24 h after surgery)
4. Life-threatening event (including cardiopulmonary crisis or anaphylaxis)
5. Readmission to the hospital any time after discharge after the procedure through the end of follow-up, owing to a complication related to the minilaparotomy procedure
6. Death or complication resulting in death occurring within 42 days of the surgery related to the minilaparotomy procedure

Secondary objectives and their outcomes include comparison of the following:

1. The safety of tubal ligation by minilaparotomy provided by COs versus AMOs as measured by AE rates (major and minor) at different points in time (intraoperatively, immediately postoperatively, and at each follow-up visit)
2. The performance (e.g., procedure times, difficulties performing the procedure, inability to complete the procedure, need for assistance to complete the procedure, and maximum reported pain experienced by the participant during the procedure) of COs versus AMOs for conducting tubal ligation by minilaparotomy
3. Participant satisfaction with the tubal ligation conducted by COs versus AMOs
4. The self-efficacy of COs versus AMOs in performing tubal ligation by minilaparotomy, defined by provider confidence, comfort, and perception of their ability to perform the procedure

Detailed definitions of the primary and secondary outcomes, including the domain (name of the outcome), specific measurement, metric, method of aggregation, and time point for measurement can be found in Table 1.

**Table 1:** Definitions and statistical methods for analyzing primary and secondary outcomes

Outcome	Specific measurement	Metric	Method of aggregation	Time point for measurement	Method of analysis for difference between COs and AMOs
Primary outcome					
Major AEs	AEs graded according to predefined criteria (*see* Additional file 2, Adverse event listing).	Presence of major AEs	Five-level ordinal categorical measure of AE severity, grouped as major (IIIa–V) or minor (I and II)	During the tubal ligation procedure and through 42 days of follow-up	Chi-square test
Secondary outcomes					
Major and minor AEs at different time points during the study	AEs graded according to predefined criteria (*see* Additional file 2, Adverse event listing).	Presence of major or minor AEs	Five-level ordinal categorical measure of AE severity, grouped as major (IIIa–V) or minor (I and II).	▪ Intraoperatively ▪ Immediately postoperatively ▪ 3 days postoperatively ▪ 7 days postoperatively ▪ 42 days postoperatively	Chi-square test.
Performance: procedure time	Time to event in minutes	Difference between procedure end time and start time	Continuous measure reported as mean procedure time	Intraoperatively	Independent sample *t* test
Performance: difficulties performing procedures	Yes/no	Reported occurrence	Categorical measure of proportion of occurrence		Chi-square test
Performance: inability to complete procedure	Yes/no	Reported occurrence	Categorical measure of proportion of occurrence		Chi-square test
Performance: need for assistance to complete procedure	Yes/no	Reported occurrence	Categorical measure of proportion of occurrence		Chi-square test
Performance: maximum reported pain experienced by participant during procedure	Visual analogue scale (0 = no pain, 10 = worst possible pain)	Reported level of pain	Continuous measure reported as mean pain score		Independent sample *t* test
Participant satisfaction with tubal ligation	Rating scale	Reported level of satisfaction	Four-category ordinal value (very satisfied, somewhat satisfied, somewhat dissatisfied, or very dissatisfied)	Day 3 and day 42 follow-up visits	Ordinal logistic regression
Self-efficacy of providers in performing tubal ligation by minilaparotomy	Self-administered confidence scale	Reported level of confidence	Continuous measure with higher value indicating greater level of confidence	At completion of recruitment	Independent sample *t* test
	Self-administered comfort scale	Reported level of comfort	Continuous measure with higher value indicating greater level of comfort		Independent sample *t* test
	Self-administered self-efficacy questionnaire adapted from the General Self-Efficacy Scale	Reported level of self-efficacy	Continuous measure with higher value indicating greater level of self-efficacy		Independent sample *t* test

*AE* Adverse event

### Study sites

We will conduct the study at seven sites based at health care facilities in northern Tanzania (Table 2). In addition to provision of tubal ligation services at these seven static sites, we will use outreach approaches that are currently part of the GOT’s approach to increasing access to family planning. During outreach events, the trained COs and AMOs from the seven static study sites will travel to and perform procedures at other facilities where tubal ligation is not routinely available. All seven of the static study sites, as well as the outreach sites, are currently part of the GOT family planning program, with tubal ligation by minilaparotomy normally provided by physicians who are not among the providers included in the study.

**Table 2:** Study sites

District	Static study site
Arusha City	Daraja 2 Health Centre
	Kaloleni Health Centre
	Levolosi Urban Health Centre
Karatu District	Karatu Designated District Hospital
Longido District	Longido Health Centre
Monduli District	Monduli District Hospital
	Mto wa Mbu Health Centre

### Study population and eligibility criteria

We will recruit a total of 1970 participants from among women presenting at static or outreach study sites for tubal ligation. Study staff will ask women if they are potentially interested in participating in the study after they have undergone the standard GOT family planning counseling, chosen tubal ligation as the contraceptive option of their choice, and signed the GOT informed consent documents for the tubal ligation procedure.

We will randomize women if they meet any of the following criteria:

1. Are aged 18 years or older
2. Requested and consented (in accordance with GOT procedures) to tubal ligation and additionally freely consented to participate in the study and signed a study informed consent form
3. Are of sound mind, are in good general health, and are deemed suitable to undergo tubal ligation by minilaparotomy in accordance with the GOT guidelines [21]
4. Are able to understand study procedures and the requirements of study participation
5. Agree to return to the study site for the full schedule of follow-up visits
6. Agree to provide the study staff with an address, phone number, name of a close relative, and/or other locator information while participating in the research study

We will exclude women if they meet any of the following criteria:

1. Are pregnant
2. Are between 8 and 42 days postpartum or postabortion
3. Have a known allergy or sensitivity to lidocaine or other local anesthesia
4. Take a medication that would be a contraindication for elective surgery, such as an anticoagulant or steroid
5. Have had previous abdominal or pelvic surgery
6. Have a local skin infection near the area where the incision will be made
7. Have severe anemia (regardless of type or etiology), a coagulation disorder, hypertension (properly taken measurements: systolic pressure ≥ 160 mmHg or diastolic pressure ≥ 100 mmHg), acute deep venous thrombosis/pulmonary embolism, or current ischemic heart disease
8. Have unexplained vaginal bleeding; malignant gestational trophoblastic disease; cervical, endometrial, and/or ovarian cancer; pelvic inflammatory disease (current or within the last 3 months); or current purulent cervicitis, chlamydial infection, and/or gonorrhea
9. Have current symptomatic gallbladder disease, active viral hepatitis, tuberculosis of pelvic organs, acute bronchitis or pneumonia, or systematic infection or gastroenteritis
10. Are currently participating in another biomedical research study

### Generation of allocation sequence and random allocation

We used permuted blocks within the site for the randomization to ensure similarity of groups with regard to potential confounding factors. A researcher unaffiliated with the study generated by computer the random allocation sequence centrally at EngenderHealth headquarters in New York City. Randomization is in a 1:1 ratio (minilaparotomy conducted by a CO or minilaparotomy conducted by an AMO), stratified by study site, and restricted with randomly varying block sizes of four to eight within the strata. We will recruit participants at all study sites until the total sample size has been reached. A research assistant at each site will randomize participants after screening has been conducted, a woman’s eligibility for study participation has been confirmed, and just prior to start of the minilaparotomy procedure. We will achieve random allocation concealment by using a text message service (Sealed Envelope Ltd. [www.sealedenvelope.com], London, UK). Because of the nature of the services and low availability of clinical staff at study sites, it is not possible to mask participants, coinvestigators, those assessing outcomes, or other study staff to treatment allocation.

### Rationale for the noninferiority hypothesis and sample size calculation

The primary objective of this study is to determine whether the rate of major AEs following tubal ligation by minilaparotomy conducted by trained COs is not worse by more than a minimal relevant difference than the AE rate following tubal ligation by minilaparotomy conducted by trained AMOs. This question lends itself to a noninferiority design. The choice of the noninferiority margin (i.e., the smallest clinical difference that is acceptable between the two treatments) is based on a combination of clinical judgment and statistical reasoning. In the case of major AEs following tubal ligation by minilaparotomy, data from prior trials (*n* = 12) suggest an average AE rate of 3.85% (95% CI 3.15–4.54%). After removing the one trial that is an outlier, in terms of both sample size and AE rate, we calculated the average as 1.59% (95% CI 0.90–2.28%) (Table 3).

**Table 3:** Reported adverse event rates following tubal ligation by minilaparotomy

Reference	AEs (*n*)	Sample (*n*)	AE rate		
Chowdhury and Chowdhury, 1975 [31]	7	600	1.2%		
Fongsri and McDaniel, 1979 [32]	4	900	0.4%		
Dusitsin et al., 1980 [33]	5	292	1.7%		
Koetsawang et al., 1981 [34]	37	1376	2.7%		
Satyapan et al., 1983 [35]	82	3549	2.3%		
Kanchanasinith et al., 1990 [36]	8	820	1.0%		
Jack and Chao, 1992 [8]	482	5182	9.3%		
Ruminjo and Ngugi, 1992 [6]	8	1521	0.5%		
Ruminjo and Ngugi, 1993 [5]	30	1999	1.5%		
Cisse et al., 1997 [4]	7	800	0.9%		
Kidan et al., 2001 [9]	0	148	0.0%		
Gordon-Maclean et al., 2014 [18]	11	518	2.1%		
			AE rate (weighted average)	Lower limit	Upper limit
Total	681	17,705	3.85%	3.15%	4.54%
Total excluding Jack and Chao, 1992 [8]	199	12,523	1.59%	0.90%	2.28%

*AE* Adverse event

These data suggest that an absolute rate of approximately 3% would be expected and that we would expect no greater than 5%, based on the upper limit of the 95% CI. In addition, in the clinical judgment of EngenderHealth staff and outside experts, we have determined that a noninferiority margin of 2% is appropriate because it implies that up to a 5% AE rate would be clinically acceptable. In other words, if the upper limit of the two-sided 95% CI for the difference in AE rates (CO minus AMO) lies fully to the left of the 2% noninferiority margin, we will have proved noninferiority of tubal ligation by minilaparotomy conducted by COs at a level of significance of α = 0.05.

Assuming a 3% major AE rate in the control group (AMOs), we will demonstrate noninferiority within the margin of 2% at a one-sided significance level of α = 0.05 and a power of 80% (calculated when AE rates in both arms are the same) with a sample size of 895 per arm (1790 women in total). Adjusting by 10% for loss to follow-up, protocol violations, and withdrawals, this would result in a total sample size of 1969 women, rounded to 1970.

### Data collection procedures and participant pathway

Data will be collected for all women who consent to participate using electronic case report forms (eCRFs) we developed for the study and programmed with Open Data Kit Collect (University of Washington, Department of Computer Science and Engineering, Seattle, WA, USA; https://opendatakit.org/) on Tecno PhonePad 7II tablets (TECNO Mobile, Hong Kong, China; www.tecno-mobile.com). A summary of the schedule of enrollment, interventions, and assessments is shown in Table 4. We trained study staff on data collection using the eCRFs prior to the start of recruitment. We will routinely assess the data throughout the study for inconsistencies and common errors as well as to identify missing values or missing records. We will resolve missing data to the greatest extent possible.

**Table 4:** Schedule of enrollment, interventions, and assessments

	Study period					
	Enrollment	Allocation	Postallocation			
Time point^a^	Day 0	Day 0	Day 0	Day 3	Day 7	Day 42
Enrollment						
Eligibility screen	X					
Informed consent	X					
Allocation		X				
Interventions						
Procedure by CO			X			
Procedure by AMO			X			
Assessments						
Pregnancy test	X					
Directed physical examination	X			X	X	X
Adverse events			X	X	X	X
Interview					X	X

*AMO* Assistant medical officer, *CO* Clinical officer

^a^In most cases, screening, enrollment, allocation, and the tubal ligation by minilaparotomy procedure will all be done on the same day (designated as day 0)

After informed consent has been obtained by a research assistant, a study provider will evaluate each potential participant for clinical eligibility according to the study inclusion and exclusion criteria noted above. If eligible, a research assistant will randomize participants, and the minilaparotomy procedure will be done on the same visit in most cases (or, if not, within 7 days of screening). Providers will conduct tubal ligation procedures according to the GOT guidelines [21]. We have not placed any restrictions on clinical care that a provider deems necessary for the health and well-being of study participants, although we will collect data on all relevant concomitant care and interventions provided before or during the procedure, as well as during the follow-up period.

We will ask participants to return for three scheduled follow-up visits at 3, 7, and 42 days postsurgery. A research assistant will call or text participants the day before each scheduled visit to remind them to come for follow-up. If a participant misses a visit, a research assistant will attempt to contact the participant three times to encourage him/her to come for the visit. We will give participants 5000 Tanzanian shillings (approximately $2.25) to cover time and transport cost to/from the study site for each of the three scheduled follow-up visits. A provider may schedule additional visits if clinically necessary, and we will inform participants that they should return to the site at any time if they have problems or concerns related to the procedure.

We will collect data at three time points:

1. Prior to the minilaparotomy procedure, we will record detailed information on sociodemographic characteristics, obstetric and family planning use history, and physical examination findings.
2. At the time of surgery, we will gather details of the anesthesia and procedure, including problems encountered, assistance needed, and occurrence of AEs.
3. We will record information on physical examination findings, AEs, and participants’ experience and satisfaction postsurgery; during recovery; and at 3, 7, and 42 days after surgery, as well as during any unscheduled follow-up visits.
4. At the end of the study, we will collect information from the COs and AMOs on self-efficacy, including measures of general self-efficacy, and confidence and comfort conduting minilaparotomy.

Once an individual eCRF is completed, a research assistant will encrypt the data on the tablets and upload it through a secure connection to the database located on a server in EngenderHealth’s office in Dar es Salaam, Tanzania. Detailed procedures for the collection, management, and use of the data are outlined in a study data management plan.

Providers will assess participants for the presence of AEs during the minilaparotomy procedure and postoperative period, as well as at all scheduled and unscheduled follow-up visits, and provide treatment/referral and follow-up as clinically indicated. We will collect detailed data on AEs in the eCRFs, including type, severity, relatedness to the minilaparotomy procedure, treatment, and outcome. We will report serious AEs resulting in death to the institutional review boards (IRBs) within 24 h of when the principal investigator (PI) is notified and those resulting in hospitalization within 10 days of notification of the PI.

We will also monitor for social harm events (SHEs) such as loss of privacy, stigmatization, relationship difficulties, physical or verbal abuse, and interference with gainful employment. We will help participants address any reported SHEs or refer them to available support services appropriate for the SHE. We will report SHEs potentially related to study participation to the IRBs within 10 days of when the PI is notified. During the study, an independent clinical trial monitor will conduct periodic on-site monitoring visits as needed to ensure that the study is being conducted and informed consent is being obtained according to the approved protocol, good clinical practice standards, and applicable regulatory guidelines, as well as to monitor recruitment and data quality.

### Statistical methods

We will develop a detailed analysis plan that covers both the final analysis and the planned interim analysis prior to initiation of the analysis. The primary outcome of the trial is major AEs within 42 days of the tubal ligation by minilaparotomy (i.e., the proportion of participants experiencing a major AE by day 42 postsurgery) in an intention-to-treat (ITT) analysis. We will conduct the primary analysis for the ITT population. The ITT population would include participants for whom the protocol is violated (i.e., participants who are randomized to have their procedure done by a CO but in fact have the procedure done by an AMO, or participants who are randomized to have their procedure done by an AMO but in fact have the procedure done by a CO) and withdrawals/discontinuations. We will also conduct a per-protocol analysis for the primary outcome if we have participants who do not receive the treatment to which they were randomly allocated.

Unlike a superiority trial, where the treatment effect is the primary parameter of interest, the interpretation of a noninferiority trial’s results depends on the location of the CI for the effect of the experimental treatment relative to the margin of noninferiority and a null effect [22]. The primary analysis will be interpreted as follows. If the whole 95% CI lies above the noninferiority margin of 2%, the experimental intervention will be declared “inferior.” If the whole 95% CI lies below the noninferiority margin, the intervention will be considered to be “noninferior” to the standard treatment. If the 95% CI includes the noninferiority margin, study results will be deemed inconclusive. Finally, if the entire 95% CI lies completely below zero, the new treatment may be considered superior to the standard treatment.

We will conduct other analyses on primary and secondary outcomes related to tubal ligation by minilaparotomy, including AE rates (overall and per time point), measures of performance (e.g., procedure times, difficulties performing the procedure, inability to complete the procedure), and participant satisfaction. We will analyze data on key demographic and reproductive variables (e.g., age, education level, marital status, occupation, ethnicity, parity) to generate a profile of study participants undergoing tubal ligation by minilaparotomy at study sites. We will analyze data on key provider-level variables to generate a profile of providers, disaggregated by provider type (CO and AMO). Methods of analysis for each secondary outcome are shown in Table 1 above.

We will include data in the analyses for all outcomes for participants who withdraw or are discontinued in the study through the time their study participation ends. We will not consider observations with missing data in the analyses and will not impute any missing data.

We have recruited a three-member data and safety monitoring board (DSMB) with no direct involvement in the study, including a surgeon experienced with tubal ligation by minilaparotomy, an epidemiologist, and a statistician. The role of the DSMB will be to deal with any ethical and safety issues that may arise during the trial and to review an interim analysis. We will conduct the interim analysis after approximately one-third of the sample has had the minilaparotomy procedure and completed the 7-day follow-up visit. At the time of the interim analyses, we will provide the DSMB with safety and study quality data (e.g., recruitment and follow-up rates, protocol violations), unmasked by treatment group.

We will ask the DSMB to give advice regarding stopping the trial if they have proof beyond doubt of an important advantage or disadvantage for one of the treatment groups and if they consider that the results are likely to affect clinical practice. For the primary outcome (i.e., major AEs), the following stopping guidelines have been proposed for the DSMB:

1. At the time of the interim analysis, the DSMB may recommend stopping the study or temporarily halting recruitment if there are significantly more major AEs in one randomization group than in the other; a difference between the two treatment arms will be considered significant if the *p* value is < 0.001 [23].
2. In the event that the interim analysis shows notably higher rates of major AEs in the study participants overall (i.e., both study arms) relative to the generally accepted AE rates following tubal ligation by minilaparotomy (e.g., a > 5% rate, which is the upper limit of the 95% CI in previously published studies), the DSMB may recommend stopping the study or temporarily halting recruitment.
3. The DSMB may also recommend stopping the study, temporarily halting recruitment, or adjusting study sites if it seems that recruitment is not proceeding at rates that will allow the study to reach its target sample size within a reasonable time frame.

If the study is stopped temporarily or permanently for any reason, we will continue to follow participants already enrolled for the planned follow-up period (and longer, if necessary, based on clinical indications). We will continue to provide enrolled participants with clinical care as necessary. According to the charter agreed to by the DSMB members before the start of the trial, their role will be as advisors to the investigators; the latter will be responsible for making decisions regarding temporarily or permanently stopping recruitment.

The PI will have responsibility for informing relevant parties (e.g., other investigators, study site personnel, IRBs, trial participants, trial registries), or ensuring they are informed, of any important protocol modifications or other information pertinent to conduct of the trial via email or phone or in person in a timely fashion, depending on the nature and urgency of the information.

## Discussion

If no major safety or other issues are identified, the data from this trial may facilitate changes in the GOT regulations, allowing appropriately trained COs to provide tubal ligation by minilaparotomy. We have reason to be optimistic that COs will be able to safely perform tubal ligations by minilaparotomy. As mentioned previously, two recent systematic reviews and results of two recent small-scale studies provide data supporting the safety of task-shifting tubal ligation by minilaparotomy to nonphysician clinicians [16–19]. Moreover, surgical task-shifting initiatives have been reported to be successful in various African countries, including Malawi [24], Mozambique [25–27], and Tanzania [28], with COs and nurses demonstrating success similar to that of their higher-level counterparts in performing cesarean deliveries and other major surgical procedures more complex than tubal ligation by minilaparotomy.

If our results demonstrate that tubal ligation by minilaparotomy provided by COs is noninferior to provision by AMOs, leading to revised guidelines by the GOT and training of COs to provide tubal ligation by minilaparotomy in Tanzania, access to a very effective permanent method of contraception would increase dramatically for thousands of women, given the more widespread availability of COs at health facilities around the country relative to physicians. Three-fourths of the population of Tanzania lives in rural areas; yet, only 31% of physicians are stationed in rural areas, in contrast to 72% of COs [29]. The need for female surgical contraceptive services in Tanzania is critically urgent. If current trends continue, the number of women of reproductive age (15–49 years old) choosing female sterilization by 2020 will be more than 65,000 annually, almost a doubling of demand since 2011 [30].

Positive findings may have broader implications; task shifting to provide long-term contraceptives, if proven safe, may be an effective approach to increasing contraceptive access in many low- and middle-income countries. The results of our large-scale RCT will provide definitive data to support or refute provision of tubal ligation by minilaparotomy conducted by nonphysician clinicians and may lead to changes in government regulations in a variety of countries.

Following completion of the study, we will submit a report to United States Agency for International Development/Tanzania and to the GOT. Additionally, we will prepare policy and technical briefs for dissemination to a wide audience, dependent on the outcome of the study. Regardless of study outcomes, the research team will hold dissemination meetings with key district, regional, and national stakeholders in Tanzania. Given the likely wide potential interest and importance of the results in the international family planning community, we will also disseminate the results in at least one publication in a peer-reviewed journal and at one or more international scientific conferences. We will follow the authorship guidelines of the International Committee of Medical Journal Editors, whereby each author is required to (1) have made substantial contributions to the conception or design of the work or to the acquisition, analysis, or interpretation of data for the work; (2) have made substantial contributions to drafting the work or revising it critically for important intellectual content; (3) approve the final version to be published; and (4) agree to be accountable for all aspects of the work in ensuring that questions related to the accuracy or integrity of any part of the work are appropriately investigated and resolved. We do not intend to use professional writers.

We do not plan to disseminate the results among research participants, because the vast majority will not be returning to the facility after their final follow-up visit is complete. Moreover, the intervention being tested is a service delivery approach and is not related to participant behaviors; thus, the communities of interest for dissemination of results are government officials, policymakers, and family planning program managers and providers.

### Trial status

Enrollment began on 6 December 2016 and was completed on 16 June 2017. Follow-up was completed on 28 July 2017.

## Additional file

Additional file 1: Standard Protocol Items: Recommendations for Interventional Trials (SPIRIT) checklist. (DOC 124 kb)

## Abbreviations

AE: Adverse event
AMO: Assistant medical officer
CO: Clinical officer
DSMB: Data and safety monitoring board
eCRF: Electronic case report form
GOT: Government of Tanzania
IRB: Institutional review board
ITT: Intention to treat
MO: Medical officer
PI: Principal investigator
RCT: Randomized controlled trial
SHE: Social harm event
SPIRIT: Standard Protocol Items: Recommendations for Interventional Trials
WHO: World Health Organization
